# Color Attributes of Colored-Yarn Mixed Woven Fabrics Made of Raw-White Warps and Multicolored Wefts and Based on Weft-Backed Structures

**DOI:** 10.3390/polym10020146

**Published:** 2018-02-05

**Authors:** Tao Hua, Lau Yiu Tang, Wing Yan Chiu, Xiao Tian

**Affiliations:** Institute of Textiles and Clothing, The Hong Kong Polytechnic University, Hung Hom, Kowloon, Hong Kong, China; lauyiu.tang@connect.polyu.hk (L.Y.T.); brandicechiu@gmail.com (W.Y.C.); xiao.tian@polyu.edu.hk (X.T.)

**Keywords:** color attributes, weft-backed structure, woven fabric, raw white warp yarn, multicolored weft yarn

## Abstract

This paper reports the development of colored-yarn mixed woven fabrics by using raw white warps and multicolored-wefts, as well as a study of the influential factors on the color attributes of the resultant fabrics. Weft yarns in six colors, together with the white warp yarns, were used to create a series of fabric colors. Two types of new weft-backed structures were designed to assign the desired wefts for color mixing, as well as to reduce the white warp floats on the surface and thus, the lightness of the fabric. The effects of the proportion of yarn color components, weft density, and the introduction of black weft floats on the color attributes of fabrics, were investigated. The results show that through varying the proportion of mixing yarn color components, via fabric structure, a series of mixed red-blue and green-yellow colors for fabrics are created, respectively. Colored yarn mixed fabric presents a lowered lightness after a middle regulating layer is introduced into the structure. Compared to fabrics with a lower density, higher density fabrics possess lower lightness, higher redness and blueness in the blue-red fabrics, and higher greenness and yellowness in the yellow-green fabric. The lightness of fabric lowers after adding black yarn.

## 1. Introduction

The color attributes of colored yarn mixed woven fabrics depend on the individual yarn colors as well as fabric weaves and other structural parameters. Through using multicolored warp and/or filling yarns with designed intricate structures, colorful and figured woven fabrics are commonly made using Jacquard looms. With such intricate structures and unique features, in terms of pattern, color and texture, colorful and figured woven fabrics are widely used for fashion, home furnishings and decorations.

A number of studies have been carried out to develop design methods, as well as to investigate the color and weave effects for colorful and figured woven fabrics [1]. Dawson investigated color and weave effects with small repeat sizes. He analyzed the derivation of families of color and weave effects from a family of weaves [2]. Osaki proposed a method for the development of high quality color reproduction on silk Jacquard textiles from digital color images, in which the appropriate criteria was estimated for better evaluation of the quality of color attributes of woven textiles converted from digital color images [3,4]. Gabrijelcic et al. explored the possibility of correcting color values in woven fabrics by changing constructional parameters. Three possible ways of carrying out color values corrections were presented. They also investigated the influence of yarn count and thread density on color values of woven surfaces and presented the simulations of a defined number of fabrics made of yarns with varying counts and densities, the results of the color measurements, and an analysis of the color differences. In addition, using regression methods, they determined relationships between theoretical color values with linear behavior and the spectrophotometrical color values of bicolor woven fabrics in CIELAB color space [5,6,7]. Chae et al. proposed geometrical and colorimetrical modeling for single-layered colorful woven structures with improved accuracy in color prediction [8,9,10]. In addition, the effect of neighboring color on color perception was investigated for color appearance modeling of multi-colored woven fabrics [11]. The visual assessment results revealed that the color appearance attribute most affected by neighboring colors is different for each test color: lightness for magenta, colorfulness for yellow, and hue for cyan. The three-dimensional color prediction models were further developed for single- and double-layered woven fabrics with the improved accuracy in lightness and chrome predictions [12]. Li et al. investigated the four types of primary color combinations for full-color fabric design and mixed-color chromaticity coordinates based on CIE chromaticity diagram analysis [13]. They found that the RGBCMY (Red, Green, Blue, Cyan, Magenta, and Yellow) combination can create a relative complete and large color gamut. Mathur et al. developed a geometric model combined with a color model to predict the color contribution of each precolored yarn, in terms of color attributes of each area of a Jacquard pattern, for the automation of color/weave selection in Jacquard design [14]. Ng et al. suggested an innovative layered combination design mode, and the resultant fabric structure was capable of expressing independent full-color figuring effects on sides of fabric [15]. Wang et al. proposed an innovative design method for preparing polymeric optical fiber (POF) jacquard fabric with a dynamic pattern display. The designed POF Jacquard fabrics were capable of displaying dynamic patterns with high brightness and resolution under circuit control [16]. In order to obtain good color and figure effects of fabric, multicolored warp and filling yarns with multi-layer woven structures are commonly used in colorful and figured woven fabrics, such as three colors or even six colors for warp and weft yarns, respectively. However, with the modern weaving looms, weft yarns in different colors are used easily in woven fabric production, while the preparation of warp yarn is more demanding and complicated than that of filling yarn. Therefore, it is necessary to investigate the colorful and figured woven fabrics made by raw white warps and multicolored wefts in terms of weft yarn colors, weaves and structural parameters, as well as their effects on the resultant color attributes of fabrics.

For the colorful and figured woven fabrics formed by only using raw white warps and multicolored wefts, the created colors of fabrics mainly come from the color mixing of different colored weft yarns. In this study, based on the yarn color mixing principle, weft yarns in six different colors—red, green, blue, yellow, black and white—together with the raw white warp yarn were used to create a series of fabric colors. In addition, in order to reduce the white warp floats on the fabric surface as well as the lightness of the fabric due to these floats, two types of weft-backed structures—weft-backed structure with and without a regulating layer—were designed to construct the fabric. The effects of the proportion of yarn color components for mixing, weft density and the introduction of black weft floats on the fabric surface on the color attributes of fabrics were also explored.

## 2. Experimental Details

### 2.1. Design of Weft Yarn Colors and Weft-Backed Structures

Color is one of the most important aspects for woven fabric design. The final visualized color of colorful and figured woven fabrics is the result of the combined weave color effect, achieved from a series of different colored yarns, mixed in varying proportions via the weave design. Based on the coloring principle of woven fabric, a kind of color-mixing of non-transparent color, limited warp and weft yarns, in primary colors or basic colors, are commonly mixed to produce more mixed colors [17]. Therefore, the colors of warp and weft used in the fabric have a great impact on the final color and figuring effect of woven fabric. In the color mode, RGB, red, green and blue are three primary colors, while cyan, magenta, yellow and black are four primary colors for the CMYK (Cyan, Magenta, Yellow, and Black) color mode [18,19]. These two color modes have been applied in the color design of colorful woven fabrics. On this basis, weft yarns in six different colors—red, green, blue, yellow, black and white—together with raw white warp yarn were selected to create the mixed colors for the colorful and figured woven fabrics in this study.

**Figure 1:**
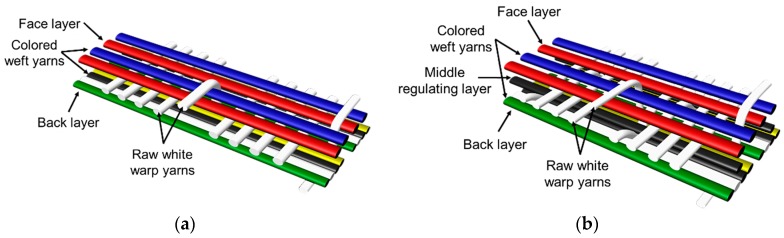
Weft-faced, weft-backed structures: (**a**) structure without regulating layer; (**b**) structure with regulating layer.

For colorful and figured woven fabrics using white warp and multicolored weft yarns, the color and figure effects of the fabric mainly depend on the arrangement of colored weft yarns for color mixing on the fabric face. Therefore, the weave design that determines the arrangement of weft yarns becomes a key point in the fabric design. In this study, weft yarns in six colors interlaced with warp yarns in white to form woven fabrics. Through the weave design, the colored weft yarn floats with white warps are arranged on the fabric face to present the color and figure of woven fabric. Based on this design concept and principle, two types of weft-faced, weft-backed structures were designed for yarn color mixing. One was a weft-backed structure, without a regulating layer, that consisted of a face layer and back layer, as shown in Figure 1a. On the basis of this structure, a modified weft-backed structure was proposed by adding a color regulating layer between face and back layers, called a weft-backed structure with a regulating layer, as shown in Figure 1b. This supplement layer in a twill weave was used to regulate the face color characteristics of fabric, reduce the color effect of back yarns on the fabric face color as well as enhance the connection between the face and back layers for an integrated and stable fabric structure. For the weft-backed woven structures with a regulating layer, the desired colored weft yarns were placed in the face layer of the fabric for color mixing, while other undesired colored weft yarns were hidden within the back layer of the fabric. Moreover, through changing the different colored yarns on the fabric surface and adjusting their proportion of yarn floats via the face weave design, a series of mixed colors could be created, to express the color and figure of woven fabrics. Table 1 presents weaves designed for weft-backed structures, with and without a regulating layer, for blue-red and green-yellow weft yarn mixings, in varied proportions on the fabric face, while weaves for mixing blue-red weft yarns with black weft yarns on the fabric face are listed in Table 2. From weave N_(BL+R)_ (1) to N_(BL+R)_ (7) and weave M_(BL+R)_ (1) to M_(BL+R)_ (7), for structures without and with a regulating layer, respectively, blue-red weft yarns were mixed in different color proportions, while green-yellow weft yarns were combined in varied color proportions using weaves N_(G+Y)_ (1) to N_(G+Y)_ (7) and weaves M_(G+Y)_ (1) to M_(G+Y)_ (7), respectively, together with the white warps. In addition, as the white warp yarn was used, a third black weft yarn was introduced into the fabric face to mix with blue-red weft yarns. Consequently, the lightness of the fabric was expected to be lowered. For each pair of N and M weaves, the face weave was designed in the same way, and the difference was that the M weaves formed the middle regulating layer for the fabric, but the N weaves did not; this allowed an investigation of the effect of the two proposed weft-backed structures on the color attributes of fabric face. 

### 2.2. Fabric Sample Preparation

In total, fifty-six woven fabric samples with mixed weft yarns in two colors and sixteen fabric samples with mixed weft yarns in two colors with black weft floats were produced using the above-mentioned colored yarns and two weft-backed structures, via a LX 3202 Staubli Jacquard machine (Staubli, Sargans, Switzerland). In the experiment, the two color mixings were red-blue mixing and green-yellow mixing, with different proportions of two colored yarns for face color, respectively. In addition to the two colors being mixed—such as blue-red mixing—the third weft yarn in black was added into the fabric face to mix with the blue and red weft yarns for three-color mixing, in order to examine changes in color strength and lightness by adding black yarns. The detailed fabric specifications are listed in Table 3. White polyester filament yarns of 100 denier (L* = 91.084; a* = −0.303; b* = 1.500) were employed for warps of all samples, and polyester filament weft yarns of 175 denier in six colors were designed and used for two or three color mixes, in varied proportions—red (L* = 41.222; a* = 57.543; b* = 34.650), green (L* = 50.175; a* = −48.791; b* = 20.356), blue (L* = 31.097; a* = 3.369; b* = −36.261), yellow (L* = 84.611; a* = −2.449; b* = 89.794), white (L* = 90.580; a* = −0.056; b* = 2.106) and black (L* = 17.390; a* = 0.015; b* = −2.668). The fabric densities of 68 picks/cm and 58 picks/cm, together with 47 ends/cm, were used for fabrics in the weft-backed structures, with and without a regulating layer, respectively. In order to investigate the effect of the weft density of the fabric face on the color attributes of the fabric, lower weft yarn densities of 49 picks/cm and 42 picks/cm were also employed to construct fabric samples using the weft-backed structures, with and without a regulating layer, respectively, in this study.

### 2.3. Characterization of Color Attributes of Fabric

The spectral reflectance values of colored yarns and the resultant color mixed fabric samples based on weft-backed structures, with and without a regulating layer, were measured using an X-rite 7000A spectrophotometer (X-Rite, Incorporated, Michigan, USA) with the following specifications: small aperture, specular excluded, and UV excluded. The colorimetric data in terms of the CIE L*, a*, b*, C*, *H*° values, based on an illuminant D65 and 10° standard observer, were calculated using reflectance measurements. Then, the resultant ∆L*, ∆a*, ∆b*, ∆C*ab and ∆H*ab and ∆*E*_CMC(2:1)_ values were also generated for the comparison of color attributes between fabric samples. The measured color attributes were compared between fabric samples in weft-backed structures, with and without a regulating layer, to exam the structure effect and weft density as well as two-color mixing and three-color mixing.

## 3. Results and Discussion

### 3.1. Effect of Proportion of Colored Yarns on Mixed Color Attributes of Fabric

**Figure 2:**
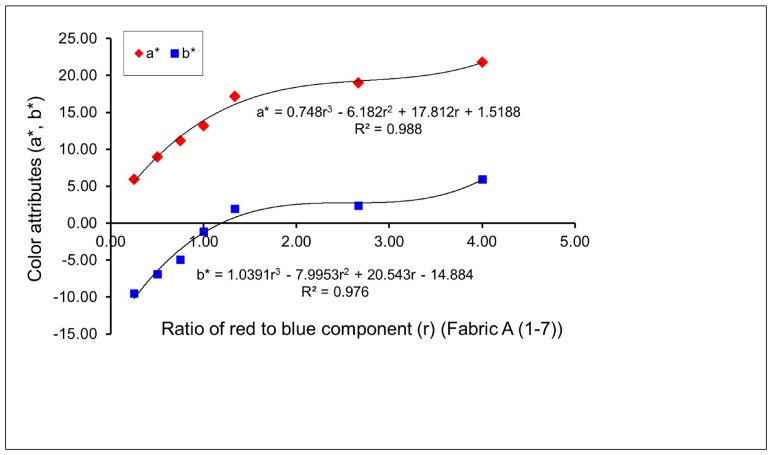
Effect of the proportion of blue-red weft yarns on mixed color attributes (a* and b*) of fabrics without a regulating layer.

**Figure 3:**
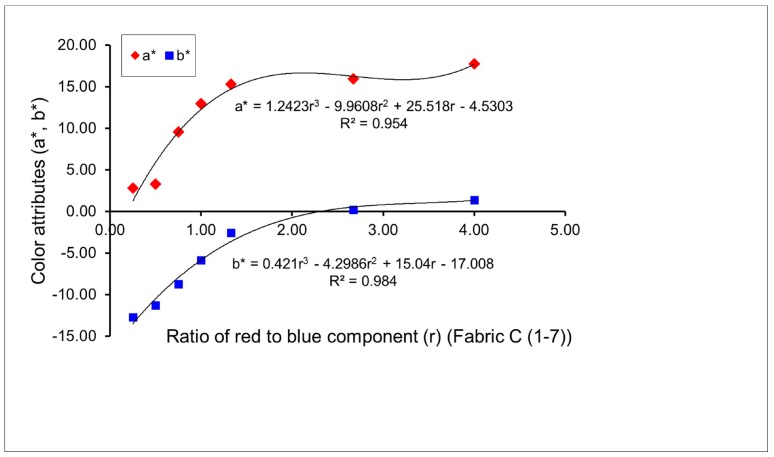
Effect of proportion of blue-red weft yarns on mixed color attributes (a* and b*) of fabrics with a regulating layer.

Four groups of twenty-eight fabric samples (A (1–7), C (1–7), E (1–7) and G (1–7)) were produced to investigate the effect of the proportion of colored yarns on the resultant mixed color attributes of fabrics. Pictures of some fabric samples are presented in Table 4. Figure 2 and Figure 3 present the color attributes (a* and b*) of fabrics in varied ratios of red to blue, without and with regulating layers, respectively. From Figure 2, it can be seen that with the gradual increase in the ratio of red to blue—from 1/4 for sample A1, to 1/2 for sample A2, to 3/4 for sample A3, to 1/1 for sample A4, to 4/3 for sample A5, to 8/3 for sample A6, to 4/1 for sample A7—the a* value increases, which means that the redness of the fabric is enhanced due to more red components being added to mix with the blue components. At the same time, the blueness of the fabric decreases, as shown by the increase in the b* value in Figure 2, when the fabric weave changes from N_(BL+R)_ (1) to N_(BL+R)_ (7). Consequently, a series of mixed blue-red colors are created. Figure 3 presents a similar trend of color attributes for fabrics C (1–7) after adding the regulating layers, wherein the redness of the fabric increases and the blueness of the fabric decreases with the red/blue ratio increase, from fabric sample C1 to C7.

**Figure 4:**
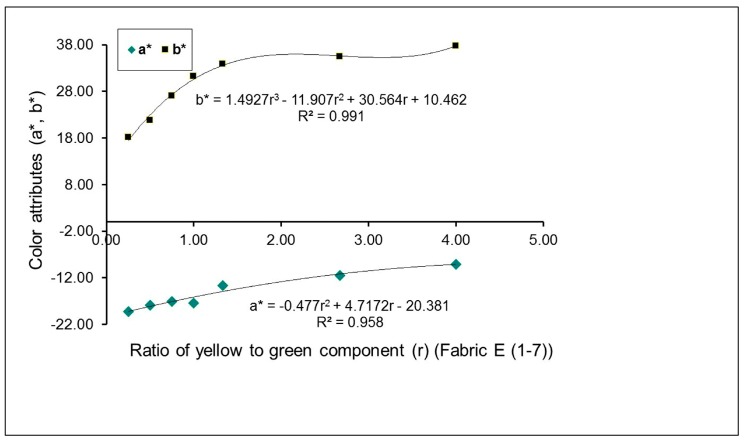
Effect of the proportion of green-yellow weft yarns on mixed color attributes (a* and b*) of fabrics without a regulating layer.

**Figure 5:**
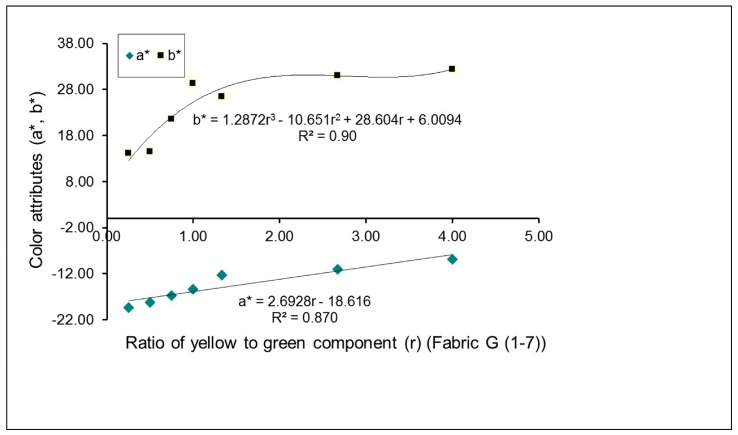
Effect of proportion of green-yellow weft yarns on mixed color attributes (a* and b*) of fabrics with a regulating layer.

The effects of the proportion of green-yellow weft yarns on mixed color attributes (a* and b*) of fabrics, with and without a regulating layer, are exhibited in Figure 4 and Figure 5, respectively. For green-yellow weft yarn mixed fabric samples without the regulating layer, E (1–7), the yellow color component increases gradually through the change in weaves, from N_(G+Y)_ (1) to N_(G+Y)_ (7). As a result, the yellowness of fabric increases while the greenness of fabric decreases, as shown by the increase in the positive a* value and the decrease in the negative b* value, in Figure 4. For green-yellow weft yarn mixed fabric samples with the regulating layer, G (1–7), it is easy to see that with an increase in the yellow component, from fabric G1 to G7, based on the transition of the corresponding weaves, M_(G+Y)_ (1) to M_(G+Y)_ (7), the positive a* value increases while the negative b* value decreases, as shown in Figure 5, indicating the deepened yellowness and lessened greenness of fabric. The pictures of fabrics (G2, G3 and G6) presented in Table 4 also demonstrate these changes.

As indicated in the previous studies, there are linear or non-linear mathematical relationships between the mixed color attributes of fabric and the proportion of yarn color components on the fabric surface [7,13]. From Figure 2, Figure 3, Figure 4 and Figure 5, it is easy to see that most combinations of red-blue and green-yellow have non-linear relationships between the mixed color attributes (a* and b*) of fabrics and the ratio of two yarn color components, for the surface color mixing of fabrics A (1–7), C (1–7), E (1–7) and G (1–7), while the a* value of fabric G (1–7) presents a linear relationship with the ratio of yellow to green components of yarns.

### 3.2. Effects of Weft-Backed Structure on Mixed Color Attributes of Fabric

As mentioned above, in order to lower the lightness of a fabric surface, due to the white warps used, a middle regulating layer was constructed in the modified weft-back fabric, wherein the black wefts and white warps interlace in a twill weave to form the middle regulating layer; this was compared to a weft-backed structure without a regulating layer. Based on such fabric structure, some black weft floats in the middle regulating layer probably appear on the fabric face through the space between the face yarns.

**Figure 6:**
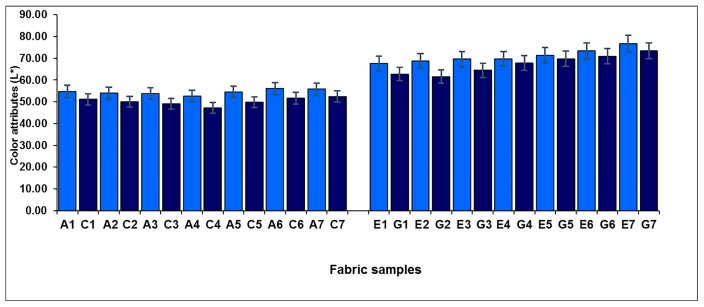
Effect of weft-backed structure on the lightness of blue-red and yellow-green mixed fabrics.

Figure 6 shows the effect of weft-backed structure on the lightness (L*) of blue-red and yellow-green mixed fabrics. It can be seen that, for each pair of blue-red mixed fabrics (A1/C1 to A7/C7), without and with regulating layers, the fabrics with regulating layers (C1 to C7) possess lower values in lightness compared to their corresponding fabrics without regulating layers (A1 to A7). For each pair of yellow-green mixed fabrics (E1/G1 to E7/G7), the fabrics show a similar trend in terms of fabric lightness, wherein the lightness of the fabric is lowered after the middle regulating layer is introduced into the weft-backed structure with black-weft floats. The pictures of a pair of fabric samples (E3/G3) are presented in Table 4. The average lightness difference (ΔL*) of blue-red mixed fabrics (A1/C1 to A7/C7) is 4.33, while the value of ΔL* of yellow-green mixed fabrics (E1/G1 to E7/G7) reaches 3.75, showing a significant difference.

**Figure 7:**
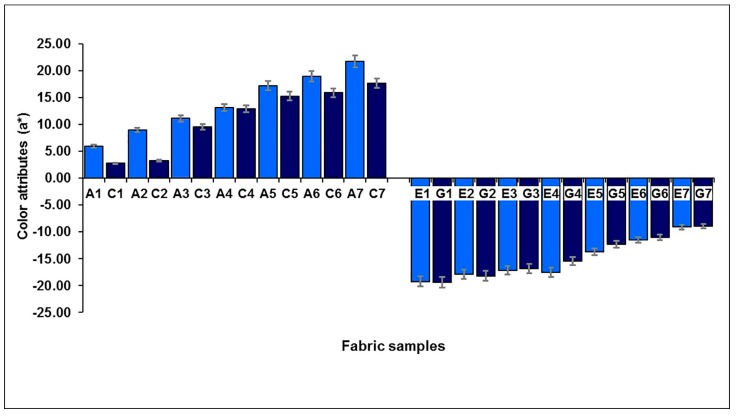
Effect of weft-backed structure on the redness and greenness of blue-red and yellow-green mixed fabrics.

**Figure 8:**
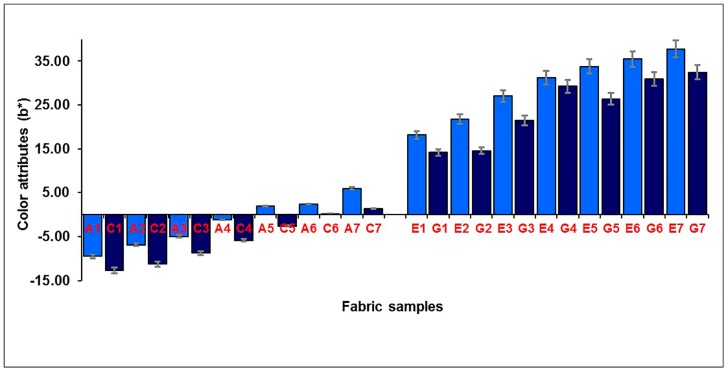
Effect of weft-backed structure on the blueness and yellowness of blue-red and yellow-green mixed fabrics.

**Figure 9:**
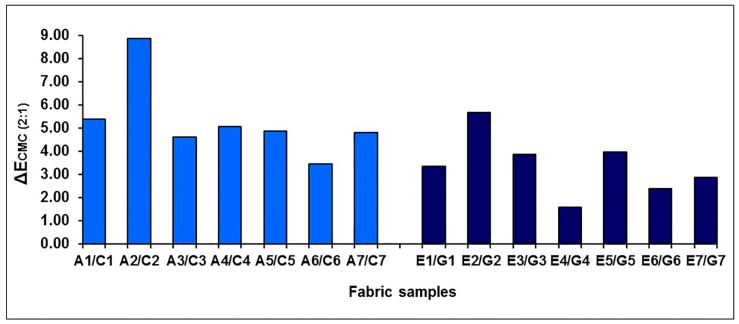
The color differences between blue-red and yellow-green mixed fabrics, without and with regulating layers.

The effect of the weft-backed structure on the redness and greenness (a*), and blueness and yellowness (b*), of blue-red and yellow-green mixed fabrics is presented in Figure 7 and Figure 8, respectively. From Figure 7 and Figure 8, it is easy to see that the blue-red mixed fabrics with regulating layers (C1 to C7) have lower redness, but higher blueness, compared to the fabrics without regulating layers (A1 to A7). The average differences in redness-greenness (Δa*) and blueness-yellowness (Δb*) of blue-red mixed fabrics (A1/C1 to A7/C7) are 2.81 and 3.92, respectively. After adding a middle-regulating layer into the structure, the yellow-green mixed fabrics with regulating layers (G1 to G7) exhibit slightly lower greenness and much lower yellowness than that of fabrics without regulating layers (E1 to E7). The average differences—Δa* and Δb*—of yellow-green mixed fabrics (E1/G1 to E7/G7) reach 0.69 and 5.14, respectively. The total color difference, ∆*E*_CMC(2:1)_, resulting from ∆L*, ∆a* and ∆b*, is from 3.47 to 8.87 with an average value of 5.31 for blue-red mixed fabrics (A1/C1 to A7/C7), and is within the range of 1.59 to 5.67 with an average value of 3.39 for yellow-green mixed fabrics (E1/G1 to E7/G7), as shown in Figure 9, indicating a significant color difference between fabrics, with and without regulating layers, based on the industrial standard for the tolerances of color difference.

### 3.3. Effects of Weft Density of Fabric on Mixed Color Attributes of Fabric

**Figure 10:**
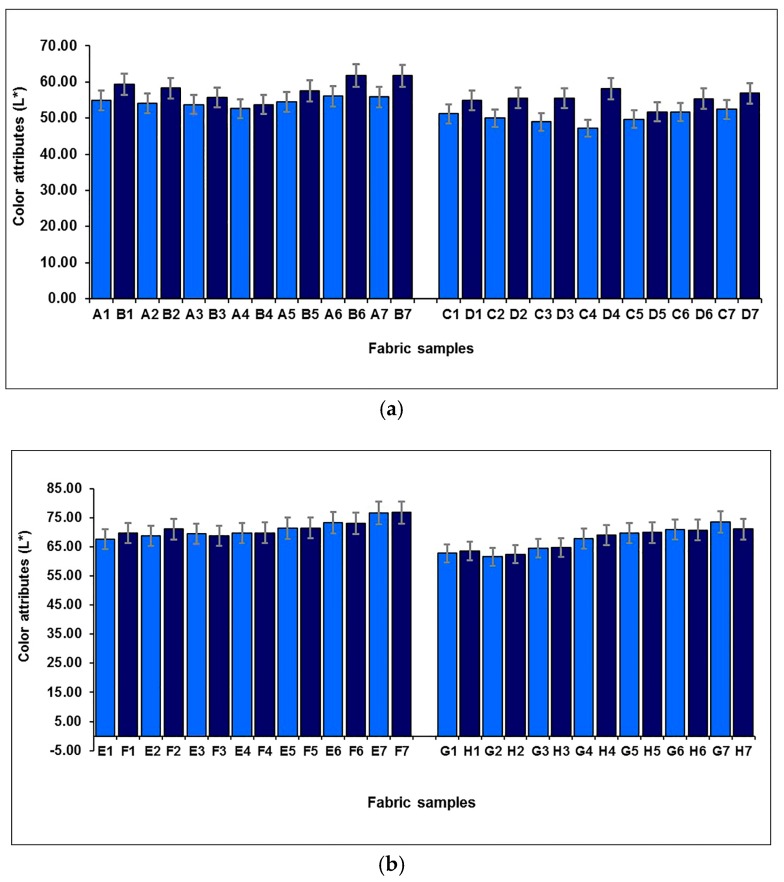
Effect of weft density on the lightness of (**a**) blue-red mixed fabrics; (**b**) yellow-green mixed fabrics.

The comparisons of lightness for each pair of higher and lower density fabrics, in blue-red and yellow-green mixing, are shown in Figure 10a,b, respectively. Pictures of some fabric samples are displayed in Table 4, including C2/D2 and C6/D6. It can be seen that both fabrics, with and without the regulating layers in a lower weft density, exhibit higher values in lightness compared to the corresponding fabrics in a higher density. One possible reason is that the lower density fabric has longer white warp floats on the fabric surface than that of the higher density fabric. In addition, more warp floats may appear from the space of face wefts, which are in the middle or back layer of fabrics, with and without the regulating layers, respectively. The average lightness differences (ΔL*) between higher and lower blue-red mixed fabrics, without and with regulating layers, reach 3.77 and 5.25, respectively, while the values of ΔL* of yellow-green mixed fabrics (E1/F1 to E7/F7) and (G1/H1 to G7/H7) are 0.84 and 0.83, respectively. The yellow-green mixed fabric shows a lower lightness difference than that of the blue-red mixed fabric. This could be explained by the yellow and green wefts possessing much higher lightness than that of the blue and red weft yarns; thus, the change in white warp floats on yellow-green mixed fabrics due to the weft yarn density does not bring a more significant effect on the fabric lightness, compared to the blue-red mixed fabrics.

**Figure 11:**
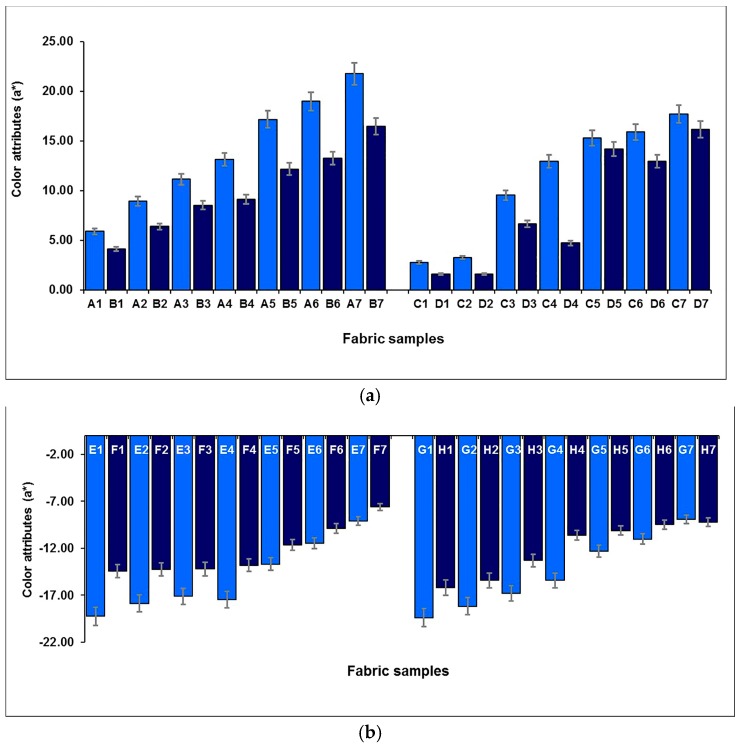
Effect of weft density on the redness and greenness of (**a**) blue-red mixed fabrics; (**b**) yellow-green mixed fabrics.

**Figure 12:**
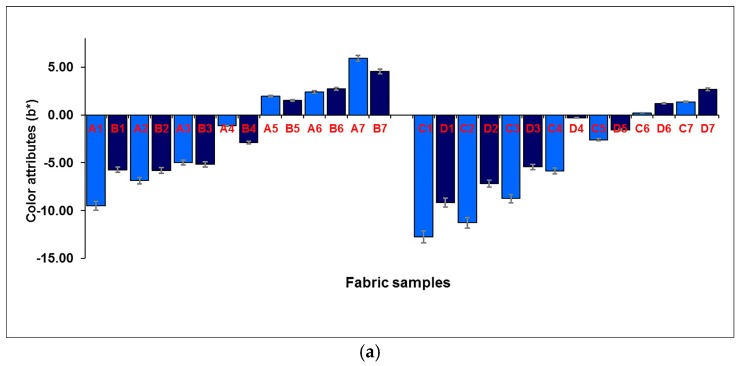

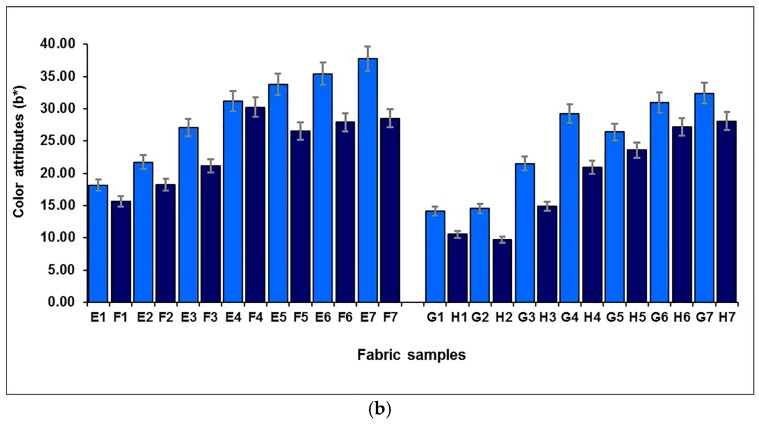
Effect of weft density on the blueness and yellowness of (**a**) blue-red mixed fabrics; (**b**) yellow-green mixed fabrics.

**Figure 13:**
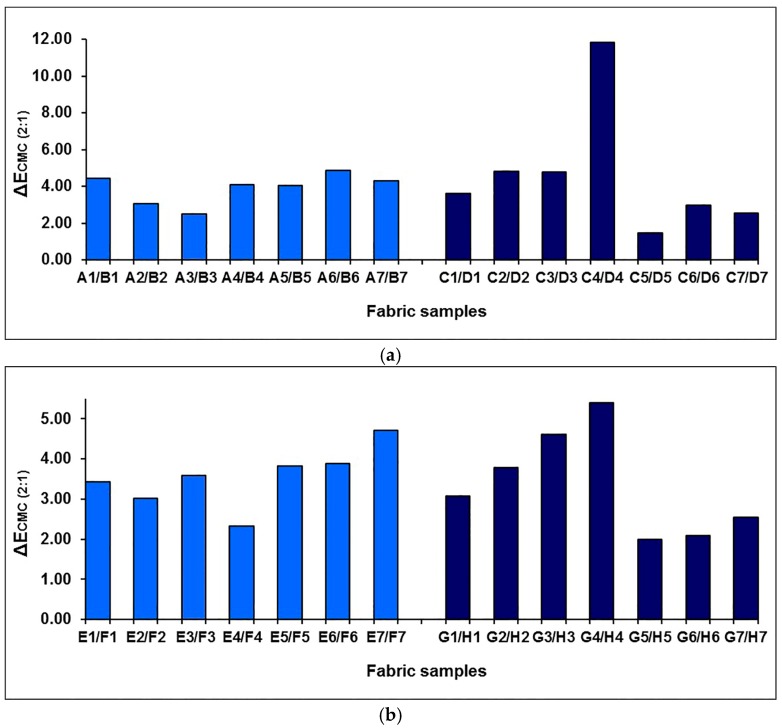
The color differences between lower and higher weft density: (**a**) blue-red mixed fabrics; (**b**) yellow-green mixed fabrics.

Figure 11 shows the comparison of each pair of higher and lower density fabrics in terms of the redness and greenness (a*). As shown in Figure 11, the higher density fabrics exhibit higher redness in the blue-red mixed fabrics and higher greenness in the yellow-green fabrics than that of the corresponding lower density fabrics. In terms of blueness and yellowness (b*), the blue-red mixed fabrics with higher densities show high blueness fabric samples A3/B3, A4/B4, A5/B5 and A7/B7, while the yellow-green fabrics with higher densities exhibit higher yellowness compared to the lower density fabrics, as shown in Figure 12. The average redness-greenness differences (Δa*) between higher and lower density blue-red fabrics, without and with regulating layers, are 3.85 and 2.78, respectively, while the values reach 2.87 and 2.63 for higher and lower density yellow-green fabrics, respectively. The higher and lower density blue-red fabrics show average blueness-yellowness differences (Δb*) of 1.28 and 2.83, with or without a regulating layer, respectively, and the values of Δb* for yellow-green fabrics, without and with the regulating layers, are 5.28 and 4.90, respectively. The total color difference between fabric samples in fabrics with a lower or higher weft density, ∆*E*_CMC(2:1)_, can be calculated from ∆L*, ∆a* and ∆b*, which is shown in Figure 13. For the blue-red mixed fabrics, the value of ∆*E*_CMC(2:1)_ for each pair of fabrics is from 2.51 to 4.89 for samples without the regulating layer, and from 1.49 to 11.82 for fabrics with the regulating layer. After adjusting the weft yarn density for yellow-green fabrics, seven pairs of fabrics without the regulating layers exhibit color differences according to the representative values of ∆*E*_CMC(2:1)_, from 2.34 to 4.72, while the samples with regulating layers present color difference values within the range of 1.99 to 5.41, as shown in Figure 13b. From the view of industrial standards for tolerances in color difference, such results indicate that the weft density has a significant effect on color attributes for the two selected weft densities in this experiment.

### 3.4. Comparison of Mixed Color Attributes between Fabrics with and without Mixing Component of Black Yarn

Another way to reduce the lightness of the fabric is to add some black weft floats on the fabric surface to mix with other colored weft yarns as well as white warp floats. In this study, based on the weaves, N_(BL+R)_ (4) and M_(BL+R)_ (4), for blue-red mixed fabrics, new weaves—N_(BL+R+BK)_ (1–4) and M_(BL+R+BK)_ (1–4)—were designed to add black weft floats with varied proportions, for weft-backed fabrics without and with a regulating layer, respectively, wherein, from the weaves N_(BL+R+BK)_ (1) to N_(BL+R+BK)_ (4) and M_(BL+R+BK)_ (1) to M_(BL+R+BK)_ (4), the black weft floats gradually increased on the fabric surface.

**Figure 14:**
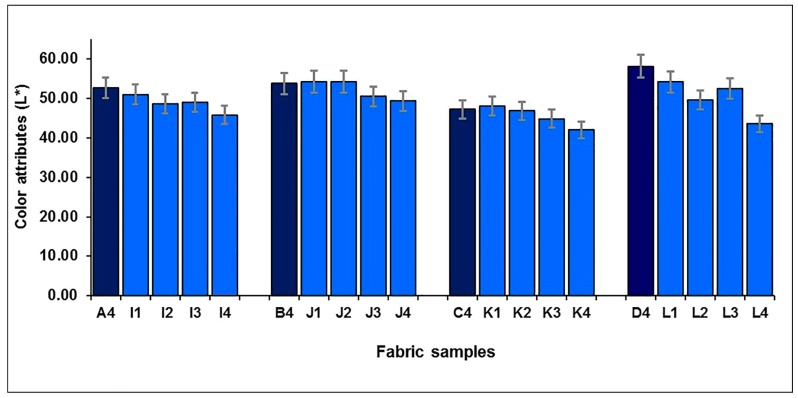
Effect of mixing black yarn on the lightness of blue-red mixed fabrics.

**Figure 15:**
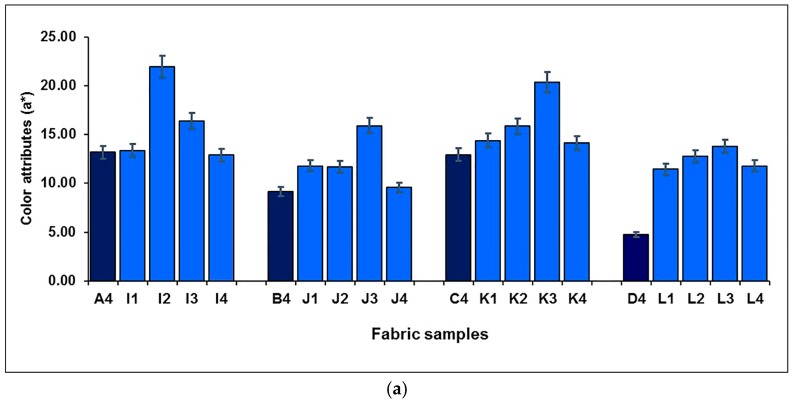

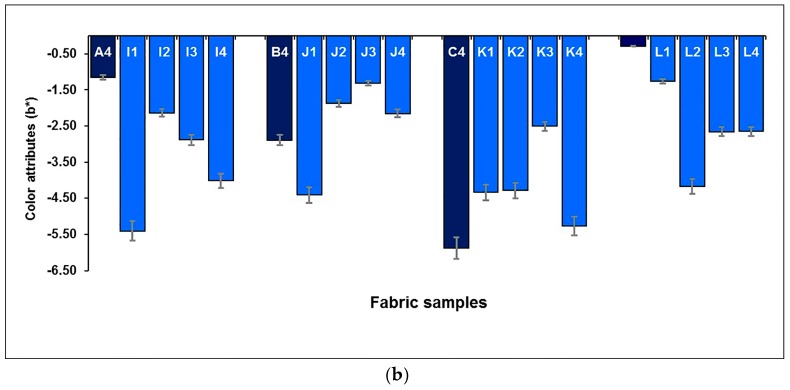
Effect of mixing black yarn on the color attributes of blue-red mixed fabrics: (**a**) redness-greenness (a*); (**b**) blueness-yellowness (b*).

Figure 14 shows the mixing component of black yarn on the lightness of blue-red mixed fabrics. It is easy to see that for both blue-red mixed fabrics, without and with regulating layers, or in lower and higher weft densities, the lightness of fabric presents a decreasing trend after adding the mixing component of black yarn. The effects of mixing black yarn on the redness-greenness and blueness-yellowness of blue-red mixed fabrics are shown in Figure 15a,b, respectively. For blue-red mixed fabrics, without and with regulating layers (I1–4, J1–4, K1–4 and L1–4), the redness-greenness (a*) increased and then decreased with the increase in black weft floats, when more black weft floats were introduced. In terms of fabric blueness-yellowness (b*), the introduction of black weft floats increased the blueness of the fabric, except for samples K1–4, but a further increase in the proportion of black yarn resulted in a decrease then increase in fabric blueness for samples I1–4, J1–4 and K1–4, as shown in Figure 15b.

**Figure 16:**
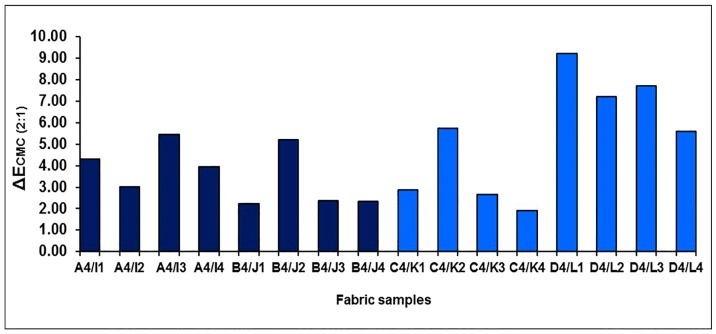
The color differences between fabrics with and without mixing component of black yarn.

The color difference, ∆*E*_CMC(2:1)_, of each pair of fabric samples is shown in Figure 16, which can be calculated from ∆L*, ∆a* and ∆b*. As shown in Figure 16, ∆*E*_CMC(2:1)_ is from 3.03 to 5.45, with an average value of 4.19, and from 2.24 to 5.19 with an average value of 3.03, for blue-red mixed fabrics without a regulating layer in a higher weft density (A4/I1 to A4/I4) and lower weft density (B4/J1 to B4/J4), respectively. It can also be seen that the average value of the color difference reaches 3.29 and 7.43 for the blue-red mixed fabrics with the regulating layer in the higher weft density (C4/K1 to C4/K4) and lower weft density (D4/L1 to D4/L4), respectively. The results indicate that fabric has a significant color difference after the black yarn is introduced.

## 4. Conclusions

In summary, two types of new weft-backed structures—weft-backed structures with and without a regulating layer—were constructed to produce colored-yarn mixed woven fabrics. By gradually increasing the ratio of red to blue and yellow to green of face wefts, a series of mixed blue-red and green-yellow colors were created, respectively. Most combinations of red-blue and green-yellow have non-linear relations between the mixed color attributes (a* and b*) of fabrics and the ratio of two yarn color components for the surface color mixing of fabrics. The fabric lightness due to the white warps used can be lowered after the middle regulating layer is introduced into the weft-backed structure with black-weft floats. The blue-red mixed fabrics with regulating layers presented lower values in redness but higher blueness, while the yellow-green mixed fabrics showed lower greenness and yellowness compared to their corresponding fabrics without regulating layers. Fabrics in a higher weft density exhibited lower values in lightness, and higher values in redness and blueness in the blue-red mixed fabrics, and greenness and yellowness in the yellow-green fabric, compared to the corresponding fabrics in a lower density. The results also show that through adding the black yarns, the lightness of fabric can be further reduced. The findings from this study can be implemented in industrial practice to produce colored-yarn mixed woven fabrics using raw-white warps and multicolored wefts, including in the design of weft yarn colors, in designing weaves for assigning colored wefts for color mixing in a varied proportion and for lowering the lightness of the fabric, and selecting proper fabric density for achieving desired color attributes.

## Figures and Tables

**Table 1:** Weaves for weft-backed structures, with and without a regulating layer, for weft yarn mixing in blue (BL) and red (R) colors, and in green (G) and yellow (Y) colors, and their varied proportions on the fabric face.

Weave code	Weft-backed structure without regulating layer	Weave code	Weft-backed structure with regulating layer	Weave code	Weft-backed structure without regulating layer	Weave code	Weft-backed structure with regulating layer
N_(BL+R)_(1)	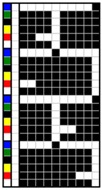	M_(BL+R)_(1)	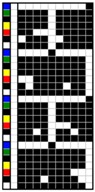	N_(G+Y)_(1)	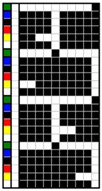	M_(G+Y)_(1)	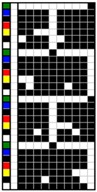
N_(BL+R)_ (2)	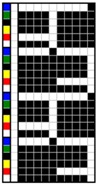	M_(BL+R)_(2)	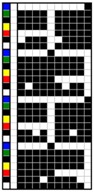	N_(G+Y)_ (2)	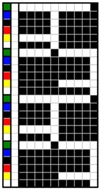	M_(G+Y)_(2)	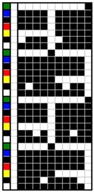
N_(BL+R)_(3)	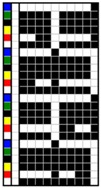	M_(BL+R)_(3)	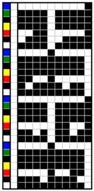	N_(G+Y)_(3)	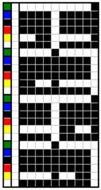	M_(G+Y)_(3)	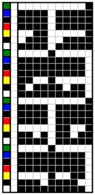
N_(BL+R)_(4)	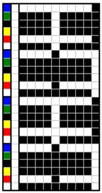	M_(BL+R)_(4)	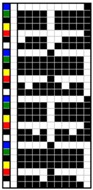	N_(G+Y)_(4)	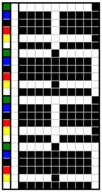	M_(G+Y)_(4)	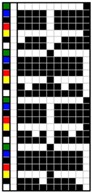
N_(BL+R)_(5)	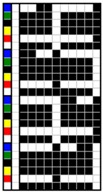	M_(BL+R)_(5)	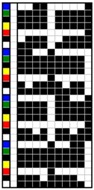	N_(G+Y)_(5)	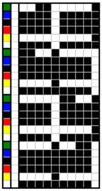	M_(G+Y)_(5)	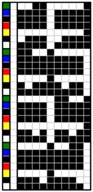
N_(BL+R)_(6)	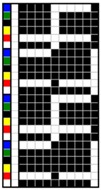	M_(BL+R)_(6)	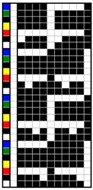	N_(G+Y)_(6)	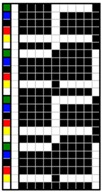	M_(G+Y)_(6)	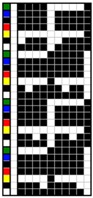
N_(BL+R)_(7)	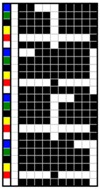	M_(BL+R)_(7)	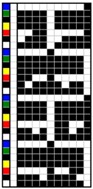	N_(G+Y)_(7)	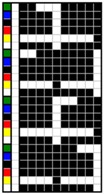	M_(G+Y)_(7)	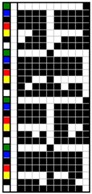

Note: M and N are used as weave codes to represent weaves for weft-backed structures with and without a regulating layer, respectively.

**Table 2:** Weaves for weft-backed structures, with and without a regulating layer, for weft yarn mixing in red (R), blue (BL) and black (BK) colors and their varied proportions on the fabric face.

Weave code	Weft-backed structure without regulating layer	Weave code	Weft-backed structure without regulating layer	Weave code	Weft-backed structure with regulating layer	Weave code	Weft-backed structure with regulating layer
N_(BL+R+BK)_(1)	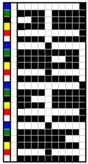	N_(BL+R+BK)_(3)	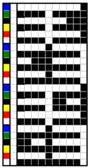	M_(BL+R+BK)_(1)	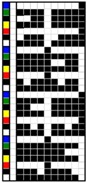	M_(BL+R+BK)_(3)	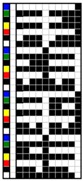
N_(BL+R+BK)_(2)	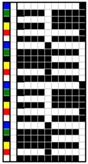	N_(BL+R+BK)_(4)	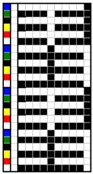	M_(BL+R+BK)_(2)	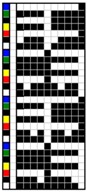	M_(BL+R+BK)_(4)	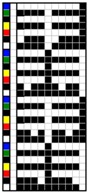

**Table 3:** Fabric specifications.

Fabric code	Yarn linear density (denier)		Weft yarn color mixing	Weave code	Fabric density (threads/cm)	
	warp	weft			warp	weft
A (1–7)	100	175	Blue + Red	N_(BL+R)_ (1–7) (without regulating layer)	47	58
B (1–7)	100	175	Blue + Red	N_(BL+R)_ (1–7) (without regulating layer)	47	42
C (1–7)	100	175	Blue + Red	M_(BL+R)_(1–7) (with regulating layer)	47	68
D (1–7)	100	175	Blue + Red	M_(BL+R)_(1–7) (with regulating layer)	47	49
E (1–7)	100	175	Green + Yellow	N_(G+Y)_ (1–7) (without regulating layer)	47	58
F (1–7)	100	175	Green + Yellow	N_(G+Y)_ (1–7) (without regulating layer)	47	42
G (1–7)	100	175	Green + Yellow	M_(G+Y)_ (1–7) (with regulating layer)	47	68
H (1–7)	100	175	Green + Yellow	M_(G+Y)_ (1–7) (with regulating layer)	47	49
I (1–4)	100	175	Blue + Red + Black	N_(BL+R+BK)_ (1–4) (without regulating layer)	47	58
J (1–4)	100	175	Blue + Red + Black	N_(BL+R+BK)_ (1–4) (without regulating layer)	47	42
K (1–4)	100	175	Blue + Red + Black	M_(BL+R+BK)_ (1–4) (with regulating layer)	47	68
L (1–4)	100	175	Blue + Red + Black	M_(BL+R+BK)_ (1–4) (with regulating layer)	47	49

**Table 4:** Pictures of fabric samples.

**Fabric picture**	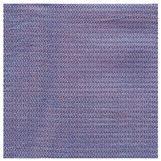	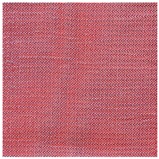	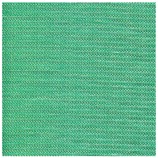	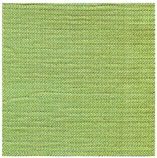
**Fabric code**	C2	C6	G2	G6
**Fabric picture**	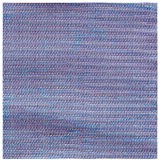	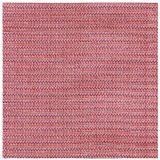	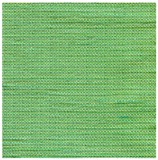	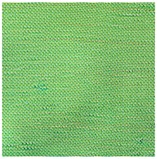
**Fabric code**	D2	D6	G3	E3
